# Correction to: Genetic diversity, distribution and domestication history of the neglected GGA^t^A^t^ genepool of wheat

**DOI:** 10.1007/s00122-021-03931-x

**Published:** 2021-08-11

**Authors:** Ekaterina D. Badaeva, Fedor A. Konovalov, Helmut Knüpffer, Agostino Fricano, Alevtina S. Ruban, Zakaria Kehel, Svyatoslav A. Zoshchuk, Sergei A. Surzhikov, Kerstin Neumann, Andreas Graner, Karl Hammer, Anna Filatenko, Amy Bogaard, Glynis Jones, Hakan Özkan, Benjamin Kilian

**Affiliations:** 1 N.I. Vavilov Institute of General Genetics, Russian Academy of Sciences, Moscow, Russia; 2 Engelhardt Institute of Molecular Biology, Russian Academy of Sciences, Moscow, Russia; 3 Independent Clinical Bioinformatics Laboratory, Moscow, Russia; 4 Leibniz Institute of Plant Genetics and Crop Plant Research (IPK), Gatersleben, Germany; 5 Council for Agricultural Research and Economics – Research Centre for Genomics & Bioinformatics, Fiorenzuola d’Arda (PC), Italy; 6 KWS SAAT SE & Co. KGaA, Einbeck, Germany; 7 International Center for the Agricultural Research in the Dry Areas (ICARDA), Rabat, Morocco; 8 Independent Researcher, St. Petersburg, Russia; 9 School of Archaeology, Oxford, UK; 10 Department of Archaeology, University of Sheffield, Sheffield, UK; 11 Department of Field Crops, Faculty of Agriculture, University of Çukurova, Adana, Turkey; 12 Global Crop Diversity Trust, Bonn, Germany

## Correction to: Theoretical and Applied Genetics 10.1007/s00122-021-03912-0

The 3rd author name contained a mistake in the original publication. The correct name should read as follows: Helmut Knüpffer

The original article has been corrected.

