# Supplementary figures and images for: Genetic diversity, distribution and domestication history of the neglected GGAtAt genepool of wheat
Source: Theor Appl Genet. 2021 Jul 20;135(3):755–76. doi: 10.1007/s00122-021-03912-0 (PMC8942905; doi:10.1007/s00122-021-03912-0)

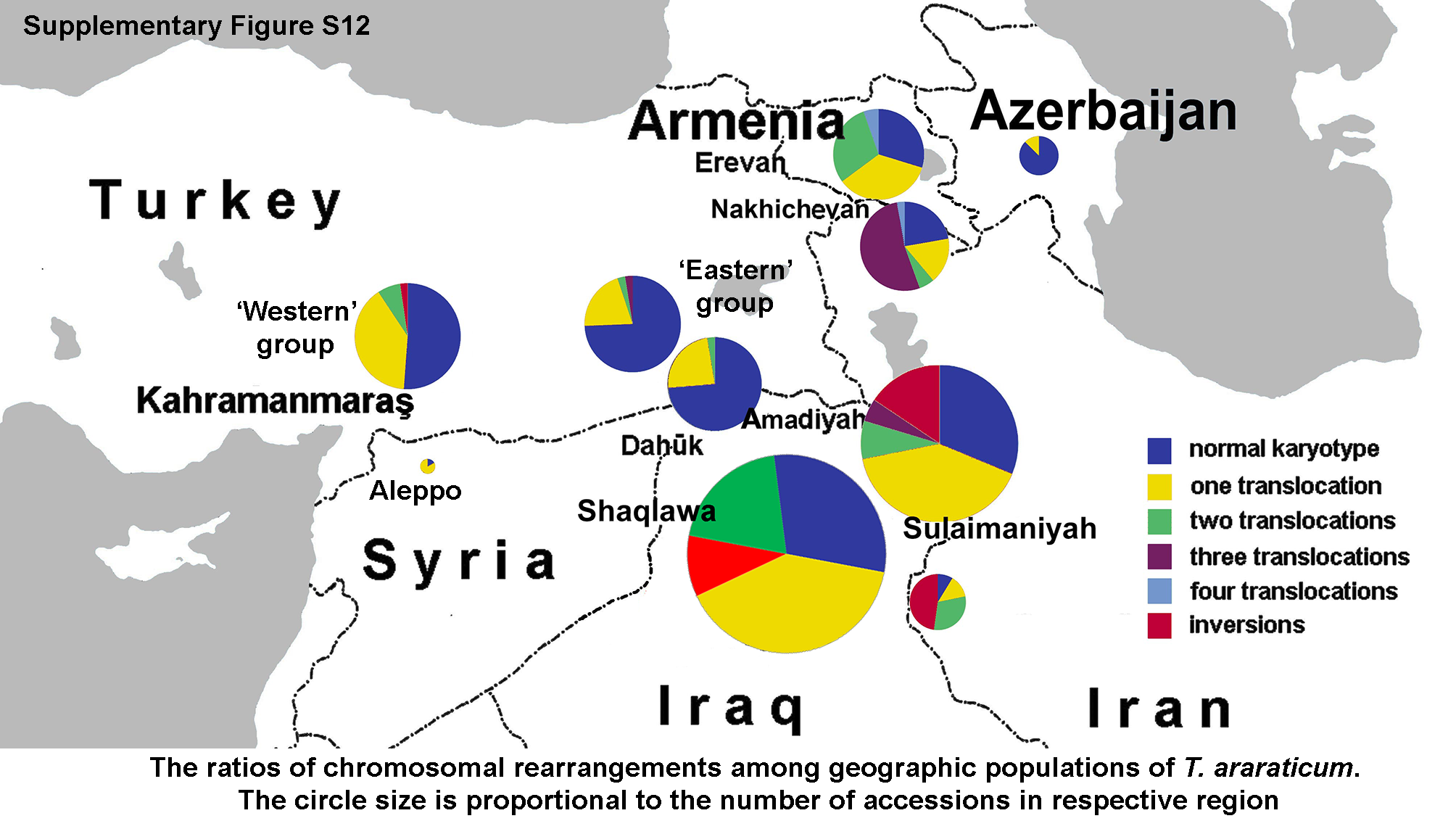

Supplement: Supplementary file 1 — Supplementary file1 (TIF 647 KB) [file 122_2021_3912_MOESM1_ESM.tif]

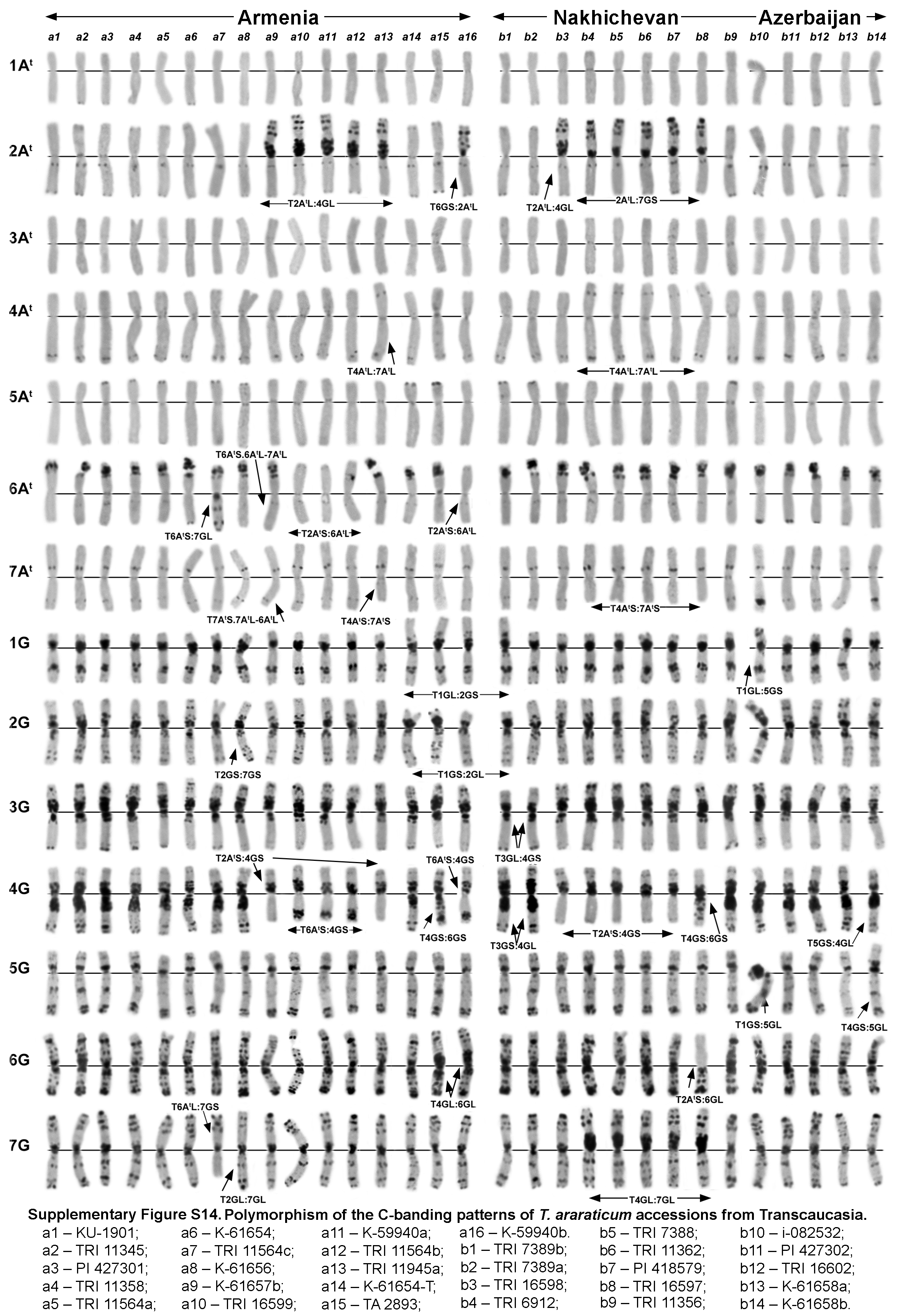

Supplement: Supplementary file 2 — Supplementary file2 (TIF 2362 KB) [file 122_2021_3912_MOESM2_ESM.tif]

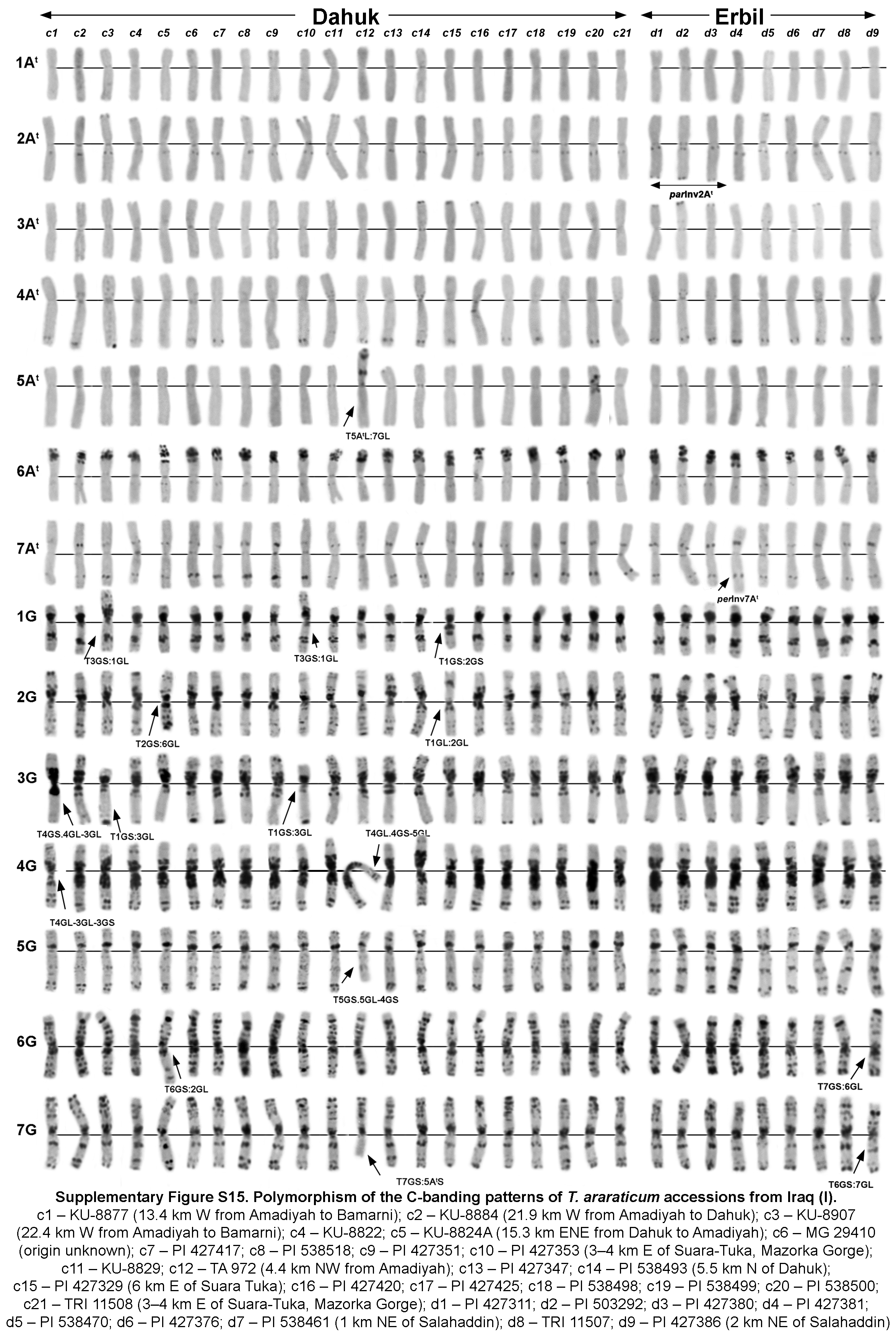

Supplement: Supplementary file 3 — Supplementary file3 (TIF 5598 KB) [file 122_2021_3912_MOESM3_ESM.tif]

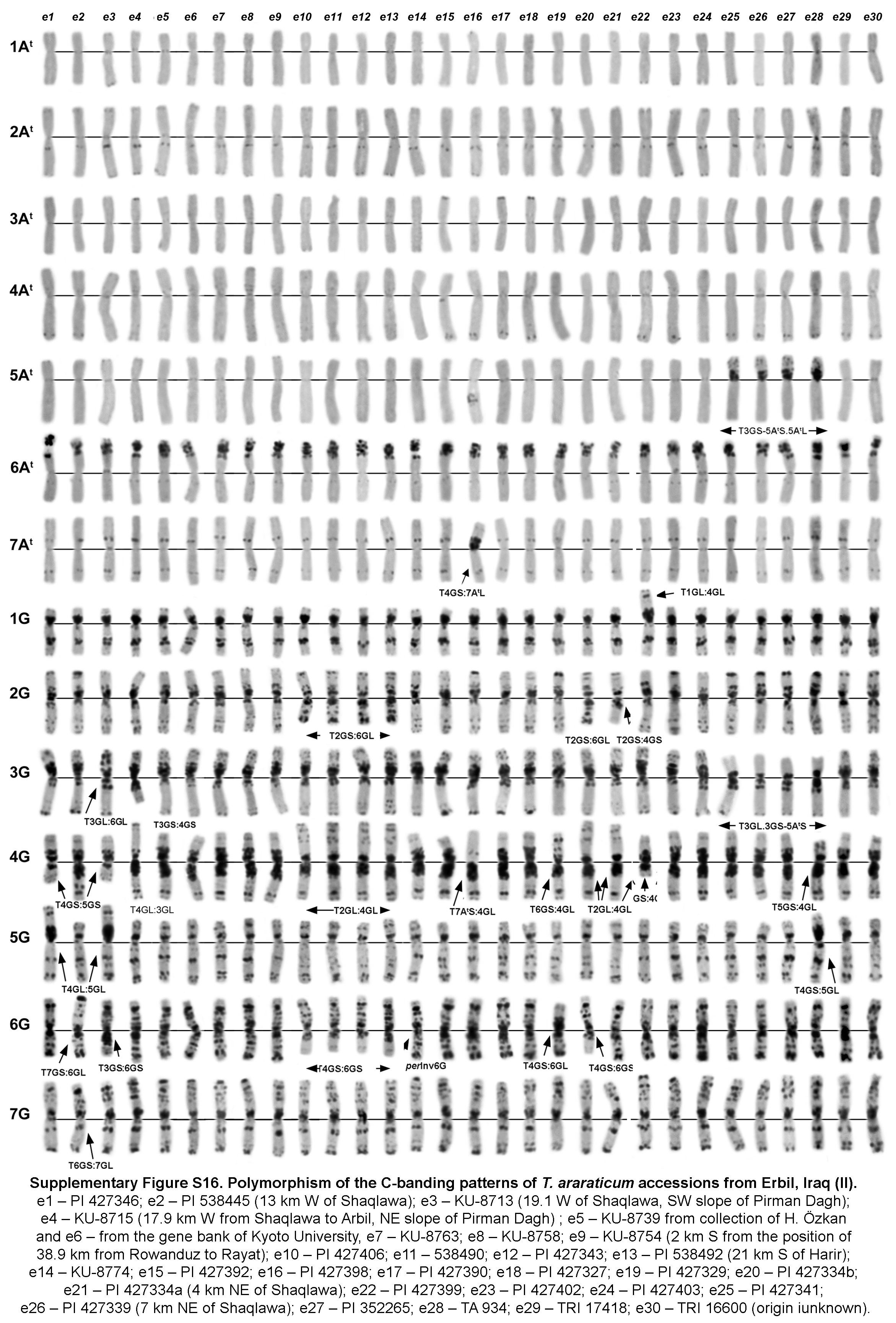

Supplement: Supplementary file 4 — Supplementary file4 (TIF 2302 KB) [file 122_2021_3912_MOESM4_ESM.tif]

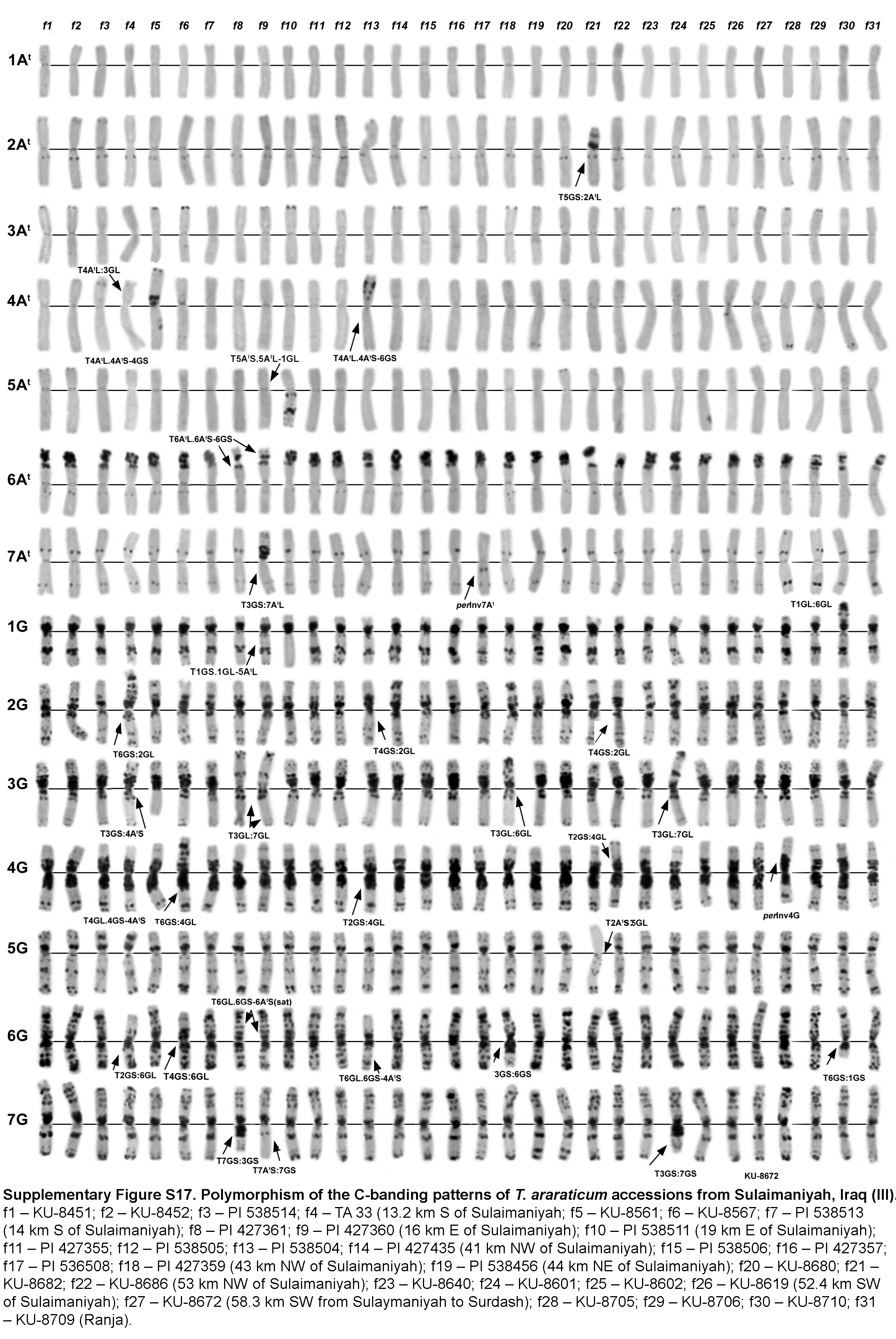

Supplement: Supplementary file 5 — Supplementary file5 (TIF 2428 KB) [file 122_2021_3912_MOESM5_ESM.tif]

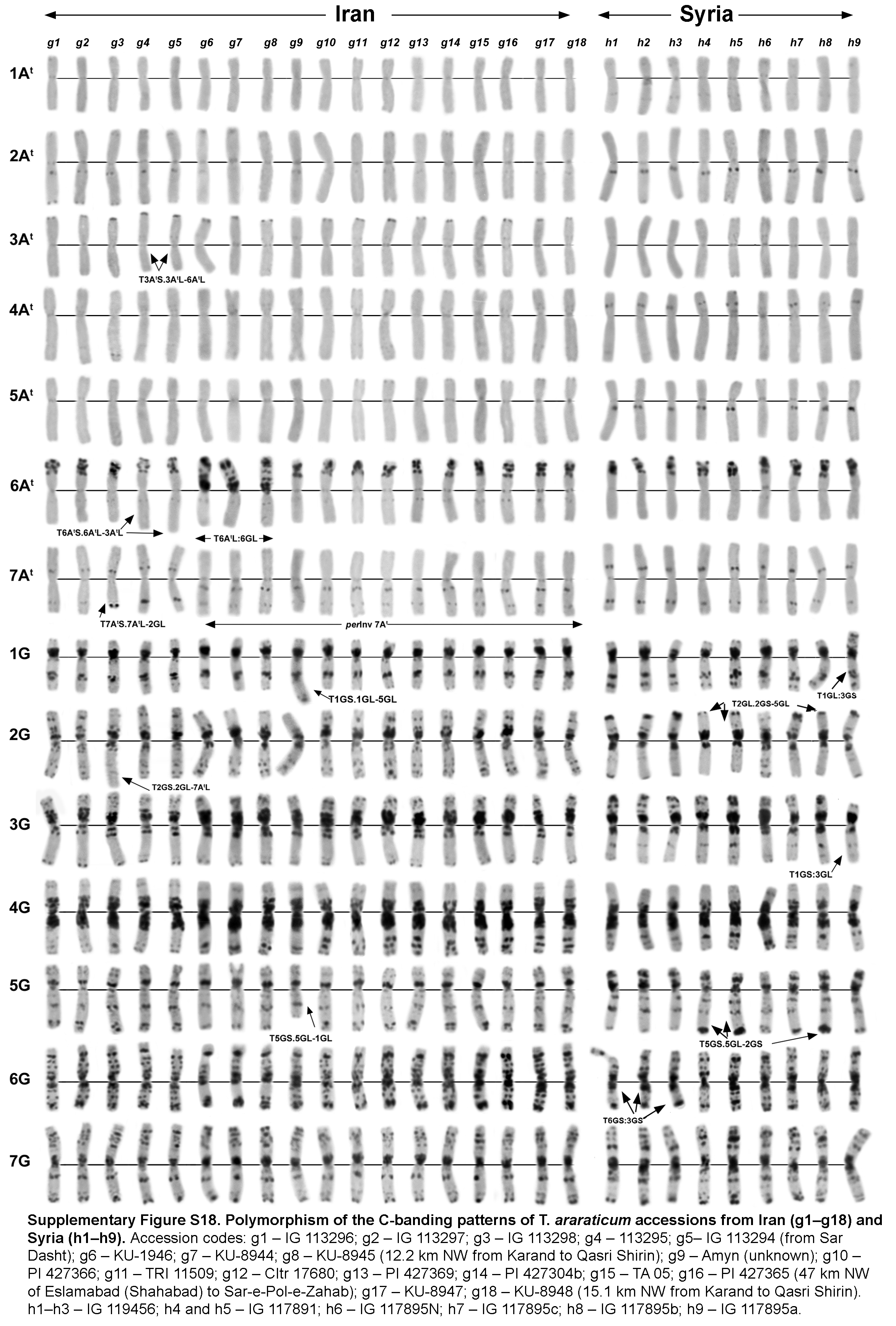

Supplement: Supplementary file 6 — Supplementary file6 (TIF 2090 KB) [file 122_2021_3912_MOESM6_ESM.tif]

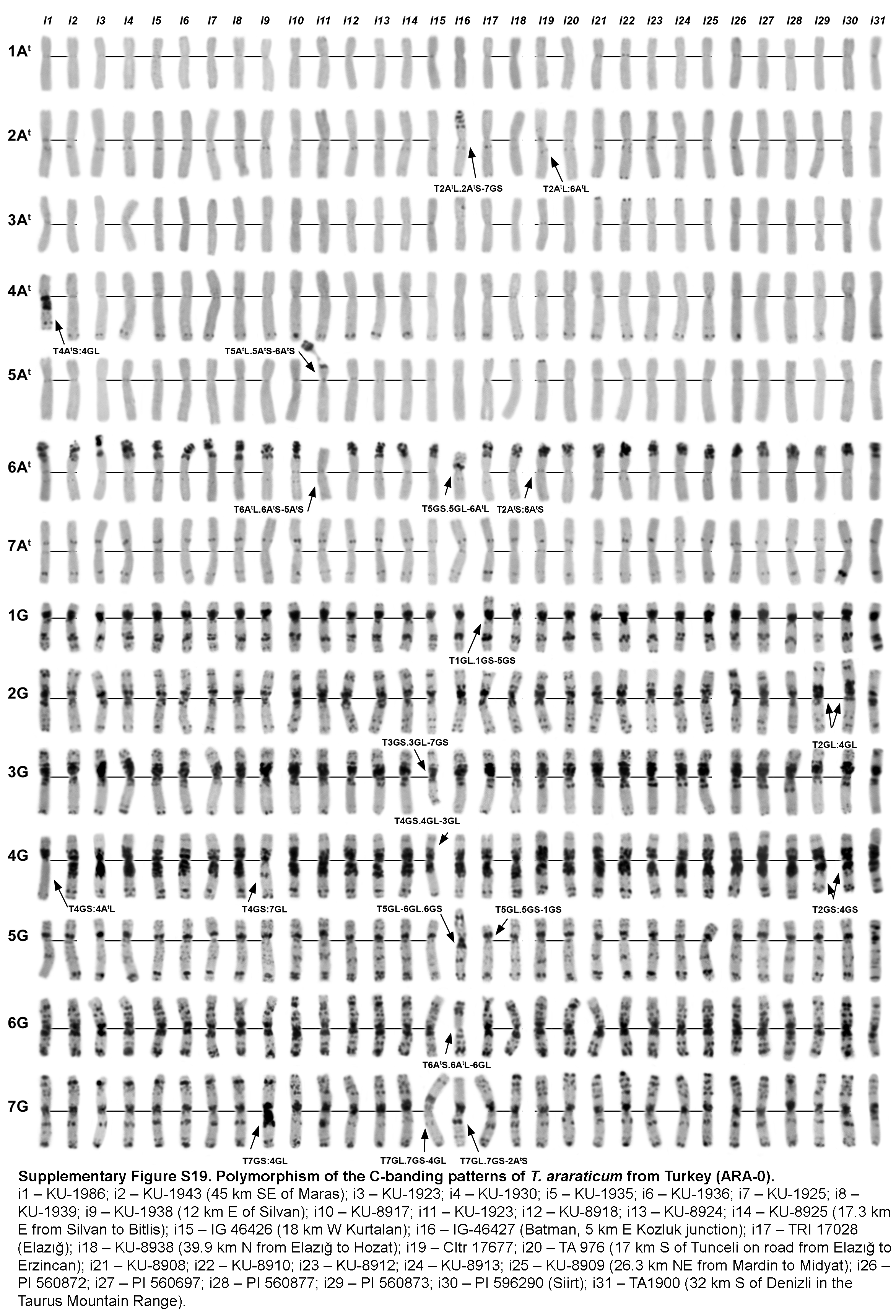

Supplement: Supplementary file 7 — Supplementary file7 (TIF 2215 KB) [file 122_2021_3912_MOESM7_ESM.tif]

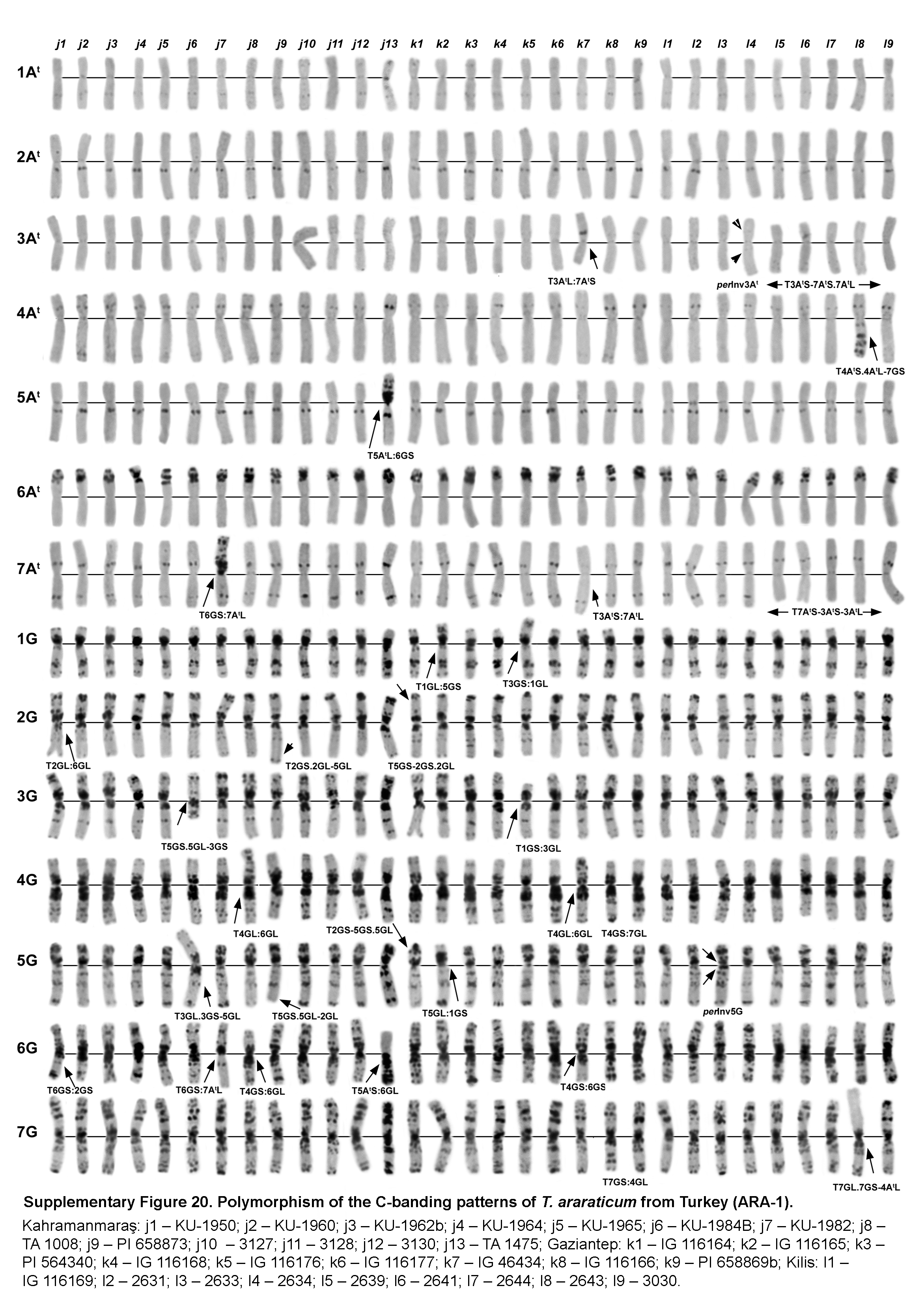

Supplement: Supplementary file 8 — Supplementary file8 (TIF 2248 KB) [file 122_2021_3912_MOESM8_ESM.tif]

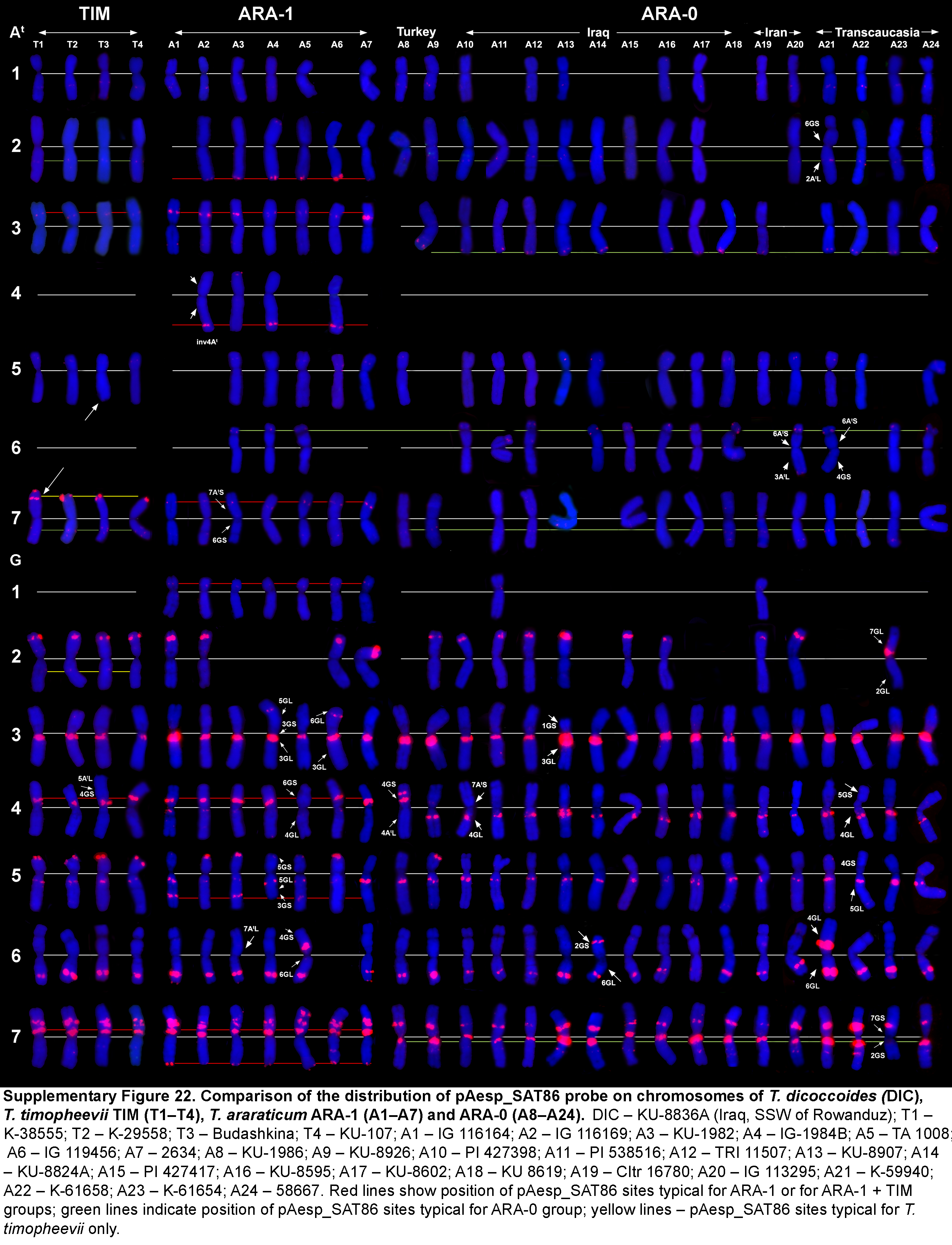

Supplement: Supplementary file 9 — Supplementary file9 (TIF 4010 KB) [file 122_2021_3912_MOESM9_ESM.tif]

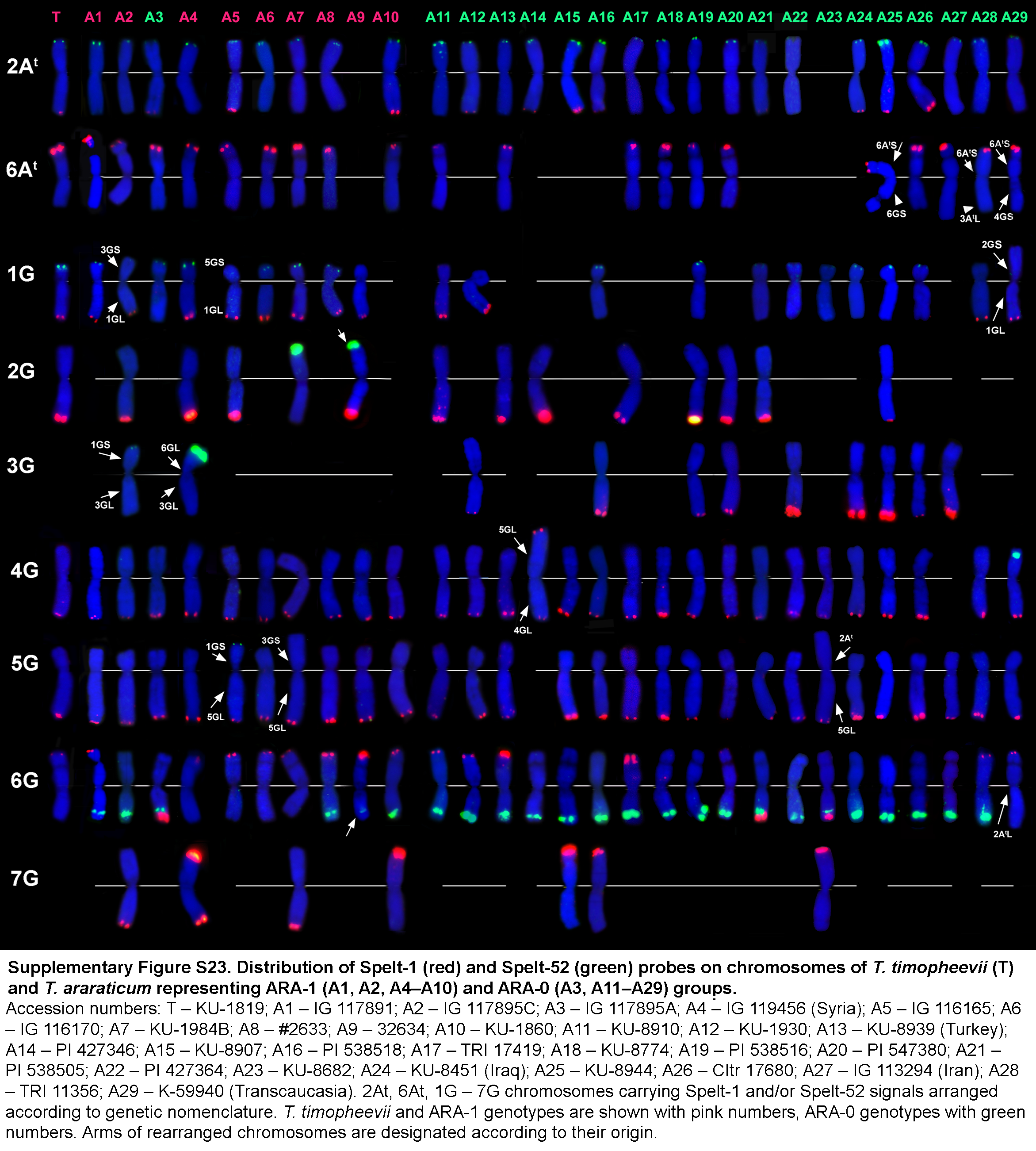

Supplement: Supplementary file 10 — Supplementary file10 (TIF 3101 KB) [file 122_2021_3912_MOESM10_ESM.tif]
