# Supplementary material for: Genetic diversity, distribution and domestication history of the neglected GGAtAt genepool of wheat
Source: Theor Appl Genet. 2021 Jul 20;135(3):755–76. doi: 10.1007/s00122-021-03912-0 (PMC8942905; doi:10.1007/s00122-021-03912-0)
Supplement: Supplementary file 11 — Supplementary file11 (DOCX 6806 KB) [file 122_2021_3912_MOESM11_ESM.docx]

**Supplementary Figure S4ssss**

**
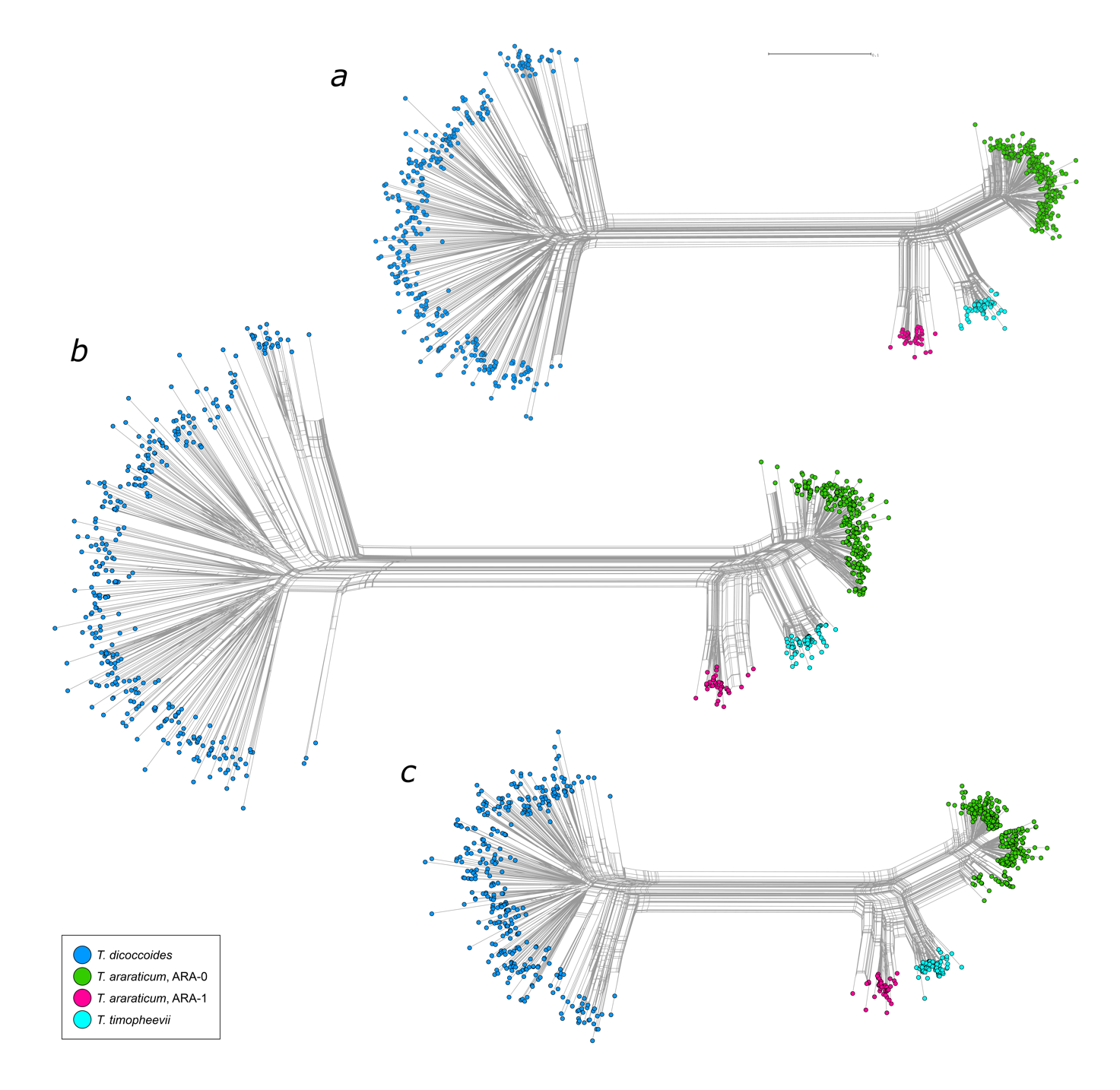
**

**NeighborNet phylogenetic networks constructed for BBAA- and GGA^t^A^t^-genome wheat accessions based on Dice genetic distances and SSAP markers.** **(a)** combined set of 656 SSAP markers (*BARE-1* and *Jeli* retrotransposon insertions); **(b)** based on 401 markers of *BARE-1* retrotransposon insertions only; **(c)** based on 255 markers of *Jeli* retrotransposon insertions only. According to our previous data (Konovalov et al. 2010) *Jeli* represents mostly the A genome, while *BARE-1* is distributed between A- and B/G-genome chromosomes.

**Supplementary Figure S5**

**
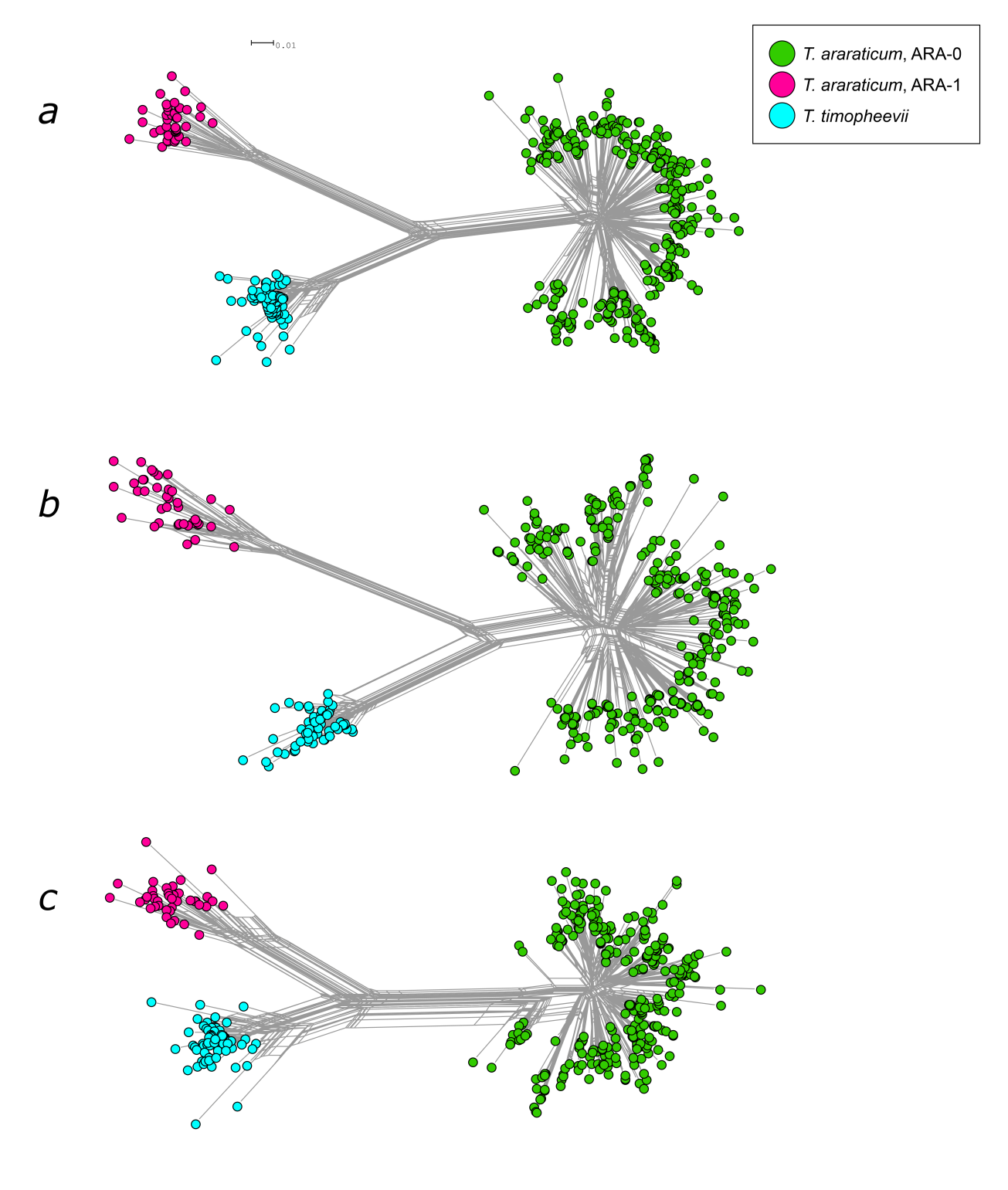
**

**NeighborNet phylogenetic networks constructed for GGA^t^A^t^-genome wheat accessions only based on Dice genetic distances and SSAP markers**. **(a)** combined set of 656 SSAP markers (*BARE-1* and *Jeli* retrotransposon insertions); **(b)** based on 401 markers of *BARE-1* retrotransposon insertions only; **(c)** based on 255 markers of *Jeli* retrotransposon insertions only. Note: the difference in distances between TIM, ARA-0 and ARA-1 groups revealed by different retrotransposon types is possibly reflecting different patterns of A^t^ and G genome diversity generated during evolution. According to our previous data (Konovalov et al. 2010) *Jeli* represents mostly the A genome, while *BARE-1* is distributed between A- and B/G-genome chromosomes.

## References

Konovalov FA, Goncharov NP, Goryunova SV, Shaturova A, Proshlyakova T, Kudriavtsev AM. Molecular markers based on LTR retrotransposons *BARE-1* and *Jeli* uncover different strata of evolutionary relationships in diploid wheats. Mol Genet Genom. 2010; <https://doi.org/10.1007/s00438-010-0539-2>
