# Supplementary material for: Genetic diversity, distribution and domestication history of the neglected GGAtAt genepool of wheat
Source: Theor Appl Genet. 2021 Jul 20;135(3):755–76. doi: 10.1007/s00122-021-03912-0 (PMC8942905; doi:10.1007/s00122-021-03912-0)
Supplement: Supplementary file 12 — Supplementary file12 (DOCX 185 KB) [file 122_2021_3912_MOESM12_ESM.docx]

**Supplementary Figure S7**

**
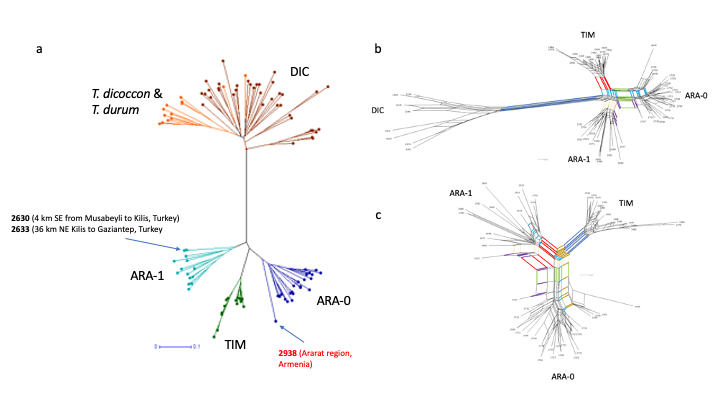
**

**Phylogenetic graphs constructed based on 146 polymorphic AFLP markers.** **(a)** Neighbor-Joining (NJ) tree for 103 genotypes based on Jaccard distances [Jaccard 1908, Perrier et al. 2003]; **(b)** NeighborNet planar graph based on Hamming distances for 59 genotypes. The following splits are highlighted: red split separating TIM; blue split separating DIC; green split separating ARA-0; creamy split separating all ARA-1; purple split – separating lines 2677 (IG 117891 from Syria; ARA-1) and 2707 (TRI 17419 from Iraq; ARA-0); light blue split – separating all TIM and four ARA-0 lines from others. **(c)** NeighborNet planar graph based on Hamming distances for 52 genotypes. The following splits are highlighted: red split – separating ARA-1; blue split – separating TIM; green split – separating ARA-0; purple split – separating lines 2677 (IG 117891 from Syria; ARA-1) and 2707 (TRI 17419 from Iraq; ARA-0); brown split – separating all TIM and four ARA-0 lines collected in Armenia and Azerbaijan; light blue split – separating all TIM, five ARA-1 and four ARA-0 lines from all other ARA-1 and all other ARA-0 lines; creamy split – separating TIM and all ARA-0 lines but line 2677 (IG 117891 from Syria; ARA-1) from all lines. See Supplementary Table S2 for more information.

## References

Jaccard P. 1908. Nouvelles recherches sur la distribution florale. Bull Soc Vaud. Sci Nat. 44:223–270.

Perrier X, Flori A, Bonnot F. (2003). Data analysis methods. In: Hamon, P., Seguin, M., Perrier, X., Glaszmann, J. C. Ed., Genetic diversity of cultivated tropical plants. Enfield, Science Publishers. Montpellier. pp 43–76.
