# Supplementary material for: Genetic diversity, distribution and domestication history of the neglected GGAtAt genepool of wheat
Source: Theor Appl Genet. 2021 Jul 20;135(3):755–76. doi: 10.1007/s00122-021-03912-0 (PMC8942905; doi:10.1007/s00122-021-03912-0)
Supplement: Supplementary file 13 — Supplementary file13 (DOCX 182 KB) [file 122_2021_3912_MOESM13_ESM.docx]

**Supplementary Figure S9**

ARA-1

ARA-0

TIM

TUR002: TA1900

**Neighbor-Joining (NJ) tree for 265 genotypes** of *T. araraticum* and *T. timopheevii* using 379 C-banding markers based on Jaccard distances (Jaccard 1908; Perrier et al. 2003).
