# Supplementary material for: Genetic diversity, distribution and domestication history of the neglected GGAtAt genepool of wheat
Source: Theor Appl Genet. 2021 Jul 20;135(3):755–76. doi: 10.1007/s00122-021-03912-0 (PMC8942905; doi:10.1007/s00122-021-03912-0)
Supplement: Supplementary file 14 — Supplementary file14 (DOCX 107 KB) [file 122_2021_3912_MOESM14_ESM.docx]

**Supplementary Material S1**

**Regarding the first finds of *T. timopheevii***

The original *T. timopheevii* accession that was used as standard in species description was obtained by P.M. Zhukovsky from Shida Kartli (Inner Kartli, formerly Gori) region of Eastern Georgia (part adjacent to Likhi Range. However, the expedition of Dekaprelevich and Menabde (1932) failed to find *T. timopheevii* in this region.

Zhukovsky described *T. timopheevii* as a wild or weedy plant in his 1923 and 1928 papers (Zhukovsky 1923, 1928), but Stoletova (1924) noted that the rachis was not as brittle as in typical wild two-grained wheats and considered the plantss found by Zhukovsky to be feral cultivated plants. She and others found *T. timopheevii* under cultivation in a restricted area of western Georgia (eastern part), mostly in the regions of Lechkhumi and Racha (Stoletova 1924–1925; Dekaprelevich and Menabde 1929, 1932; Menabde 1948, Dekaprelevich 1954). Zhukovsky's find was therefore just outside the range of twentieth century *T. timopheevii* cultivation.

**References**

Dekaprelevich LL, Menabde VL (1929) Regarding the investigation of crop plants in western Georgia. Zap. Nauchno-prikl. otdelov Tiflisskogo Bot. Sada 6:219–254 (in Russian with English summary)

Dekaprelevich LL, Menabde VL (1932). Hulled wheat in Western Georgia. Tr. prikl. bot. gen. i sel.. 5,1: 3–46 (in Russian)

Dekaprelevich LL (1954) Species, varieties and cultivars of Georgian wheat. Tr. Inst. Polevodstva AN GSSR 8:3–61 (in Russian)

Menabde VL (1948) The Georgian wheats (in Russian). Izdatelstvo Akademii Nauk Gruzinskoy SSR. Tbilisi

Stoletova EA (1924–1925) Emmer, *Triticum dicoccum* Schrank – An assay of study of one of the vanishing crops. Tr. prikl. bot. sel. 14,1:27–111 (in Russian).

Zhukovsky PM (1923) *Triticum dicoccum* Schrank *dicoccoides* Körn in Georgia. Nauchno-prikladnykh Otdelov Tiflisskogo Botanicheskogo Sada 3:1–3 (in Russian with English summary)

Zhukovsky PM (1928) A new species of wheat (in Russian with English summary). Tr. prikl. bot. gen. sel 19, (2): 59–66
